# Significant cross reactive antibodies to influenza virus in adults and children during a period of marked antigenic drift

**DOI:** 10.1186/1471-2334-14-346

**Published:** 2014-06-20

**Authors:** Michal Mandelboim, Michal Bromberg, Hilda Sherbany, Inbar Zucker, Karnit Yaary, Ravit Bassal, Rita Dichtiar, Danny Cohen, Tamar Shohat, Ella Mendelson, Manfred S Green

**Affiliations:** 1 Central Virology Laboratory, Ministry of Health, Public Health Services, Chaim Sheba Medical Center, Tel Hashomer, Ramat-Gan, Israel; 2 Israel Center for Disease Control, Ministry of Health, Gertner Institute for Health Policy Research, Chaim Sheba Medical Center, Tel Hashomer, Ramat-Gan, Israel; 3 Department of Epidemiology and Preventive Medicine, School of Public Health, Sackler Faculty of Medicine, Tel-Aviv University, Tel-Aviv, Israel; 4 School of Public Health, University of Haifa, Haifa, Israel

## Abstract

**Background:**

Little is known about the development of cross-reactive antibodies following natural exposure to pathogens. Such knowledge is critical in the development of new universal influenza vaccines.

**Methods:**

To study the possibility of the presence of cross-reactive antibodies to influenza viruses which underwent a major antigenic drift between the years 1999 and 2007 sera from samples of 80 children and 400 adults were selected at random from the Israeli national serum bank. The sera was obtained in 2002 and in 2007, two time points that followed a major drift in the infectious H3N2 influenza virus strain (A/Panama/2007/99 to A/Wisconsin/67/2005).

**Results:**

In the summer of 2002, 13% of the children had Hemagglutination Inhibition (HI) antibody titers of at least 40 and these antibodies recognized both A/Panama/2007/99 and A/Wisconsin/67/2005, where the latter strain only began to circulate in Israel in 2006. In 2007, 29% of the children had HI antibody titers of at least 40 directed against both A/Wisconsin/67/2005 and A/Panama/2007/99, even though they had never been exposed to the latter virus. Anti-A/Panama/2007/99 antibodies were detected in 58% and 68% of the 2002 and 2007 adult samples, respectively, while 8% and 39% had antibodies against A/Wisconsin/67/2005, respectively.

**Conclusions:**

The presence of naturally occurring cross-reactive influenza virus antibodies in a significant percentage of children has important implications for the development of a universal influenza vaccine.

## Background

The influenza virus is responsible for annual epidemics which result in increased primary care visits, hospitalizations, loss of work days and death, especially in the elderly and chronically ill population [1,2]. The respiratory symptoms that result from infection by influenza viruses are usually self-limiting. However, a small percentage of patients may develop primary pneumonia, which can progress to acute respiratory distress syndrome (ARDS) [3]. The combination of pneumonia and ARDS usually occurs in high-risk populations, such as those with chronic lung diseases, but also has been described in healthy individuals [4]. The majority of deaths during a seasonal outbreak occur from primary pneumonia or secondary bacterial pneumonia and excess cardiovascular disease [5].

The objective of vaccination is to induce antibodies effective against the current viruses. For many years, the seasonal vaccine has been comprised of three of the most common circulating influenza viruses, A(H1N1), A(H3N2) and B. Consequent to antigenic drift and assumption of limited cross-reactivity of antibodies against strains that are significantly different from the older strains, the virus strains included in the vaccine are “updated” to the most recent circulating viruses. When there is an antigenic shift, there could be an influenza pandemic, which can threaten the entire population due to lack of immunity against the new virus [6]. Four major pandemics occurred in the last 100 years, all resulting from influenza A infections; they included the Spanish Flu pandemic (1918–1920, H1N1) [7], the Asian Flu pandemic (1957–1958, H2N2) [8], the Hong Kong Flu pandemic (1968–1969, H3N2) [9] and the Swine Flu pandemic (2009–2010, H1N1pdm09) [10].

Influenza viruses contain eight genome segments which encode for 12 proteins [11]. Two of these, a glycoprotein named hemagglutinin (HA), and neuraminidase (NA), are expressed on the surface of the influenza virus itself and on infected cells and are involved in eliciting neutralizing antibodies against the homologous virus [12]. Therefore, both proteins are considered key targets for vaccination and are included in all types of influenza vaccines (although antibodies directed against NA are not considered neutralization antibodies). Unfortunately, the NA and particularly the HA proteins, are subject to frequent antigenic drifts and to occasional antigenic shifts. Thus the development of a universal influenza vaccine that will be effective against various influenza viruses is complex.

Antibody cross-reactivity among various influenza virus strains has been detected in several studies following immunization with influenza vaccines [13-15]. Recently, antibodies that recognize different influenza viruses have been discovered [16]. Some of these antibodies bind the HA stem region of H1, H2, H5, H6, H8, H9, H11, H12, H13, H16 influenza A viruses and others bind to the stem region of most of group H3, H4, H7, H10, H14, H15 influenza A viruses. Cross-reactive antibodies were also detected against the NA and the M proteins. Antibodies raised against the N1 subtype of human influenza viruses cross-reacted with the N1 avian influenza and partially protected mice against lethal influenza A/H5N1 virus infection [17]. Broad-reactive anti-M2 protein antibodies, raised by vaccination, provided protection against heterologous influenza virus infection in mice [18,19]. However, to the best of our knowledge, little is known about the existence of anti-influenza antibody cross-reactivity following natural exposure to seasonal influenza viruses. In this study, we examined cross-reactivity of influenza antibodies in children and adults following natural exposure to the viruses during a period of marked antigenic drift in the A(H3N2) virus.

## Methods

### Sample collection

Since the late 1990’s, serum samples have been collected, on an ongoing basis, from samples of the Israeli population, and stored frozen (-70°C) in the Israel Center for Disease Control (ICDC) repository. The adult samples were collected from individuals 35-50 years of age. The children samples were collected from 1-3-year-old children. The children analyzed in this study were not vaccinated against influenza by the time of recruitment.

### Ethics statement

The work described here is a retrospective study performed on left over samples that were obtained as part of routine tests performed. No extra samples were obtained for this research. The retrospective analysis was anonymous. Therefore, informed consent (either written or verbal) was not required. The research was approved by the Sheba Medical Center Helsinki committee (Number 94-11-12-SMC).

### Hemagglutination inhibition assay

All sera were treated with receptor destroying enzyme (RDE) (Sigma C8772), diluted 1:4, for 16 h, prior to heat inactivation (30 min, 56°C) and absorption with erythrocytes to remove non-specific hemagglutination, in accordance with a WHO-recommended protocol [20].

Serially two-fold dilutions (1:20–1:2560) of sera in 25 μl PBS were prepared in V-shaped well plates, and an equal volume of four hemagglutinin (HA) units of viral antigen were added. The mixture was then incubated at room temperature for 1 h. Fifty microliters of 0.5% chicken erythrocytes suspended in PBS were added to the wells, and mixed by shaking the plates on a mechanical vibrator. Agglutination patterns were read after 30 min and the HI titer was defined as the reciprocal of the last dilution of serum that fully inhibited hemagglutination. The cut-off value selected for a positive result was 1:40. The influenza antigens were supplied by the WHO. The following viruses were tested: A/Panama/2007/99(H3N2) active in 2001–2002 and A/Wisconsin/67/05 (H3N2), active in the 2006–2007 season.

### Data analysis

**Figure 1:**
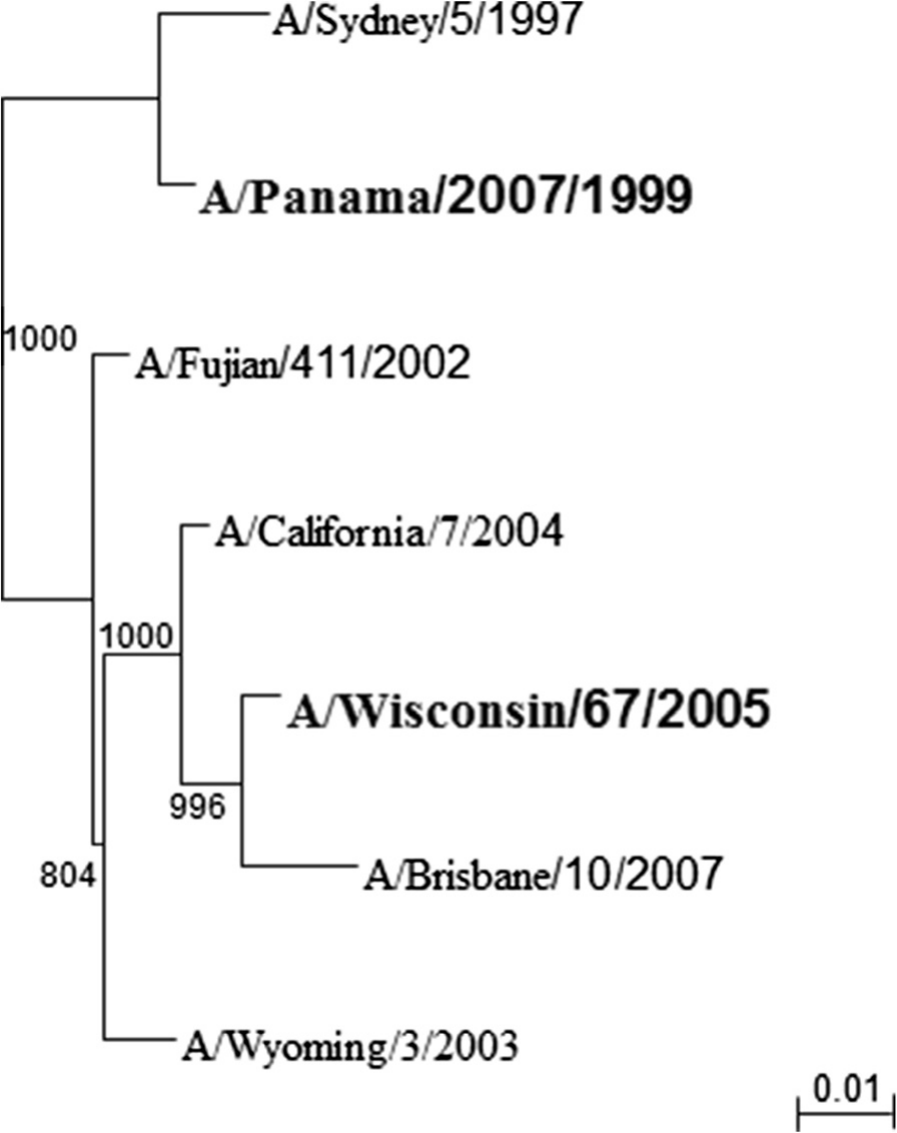
**Phylogenetic tree of the viruses evaluated in this study.** The two antigenically different viruses are highlighted in bold. 1701 nucleotides of the HA protein of each virus were compared.

**Figure 2:**
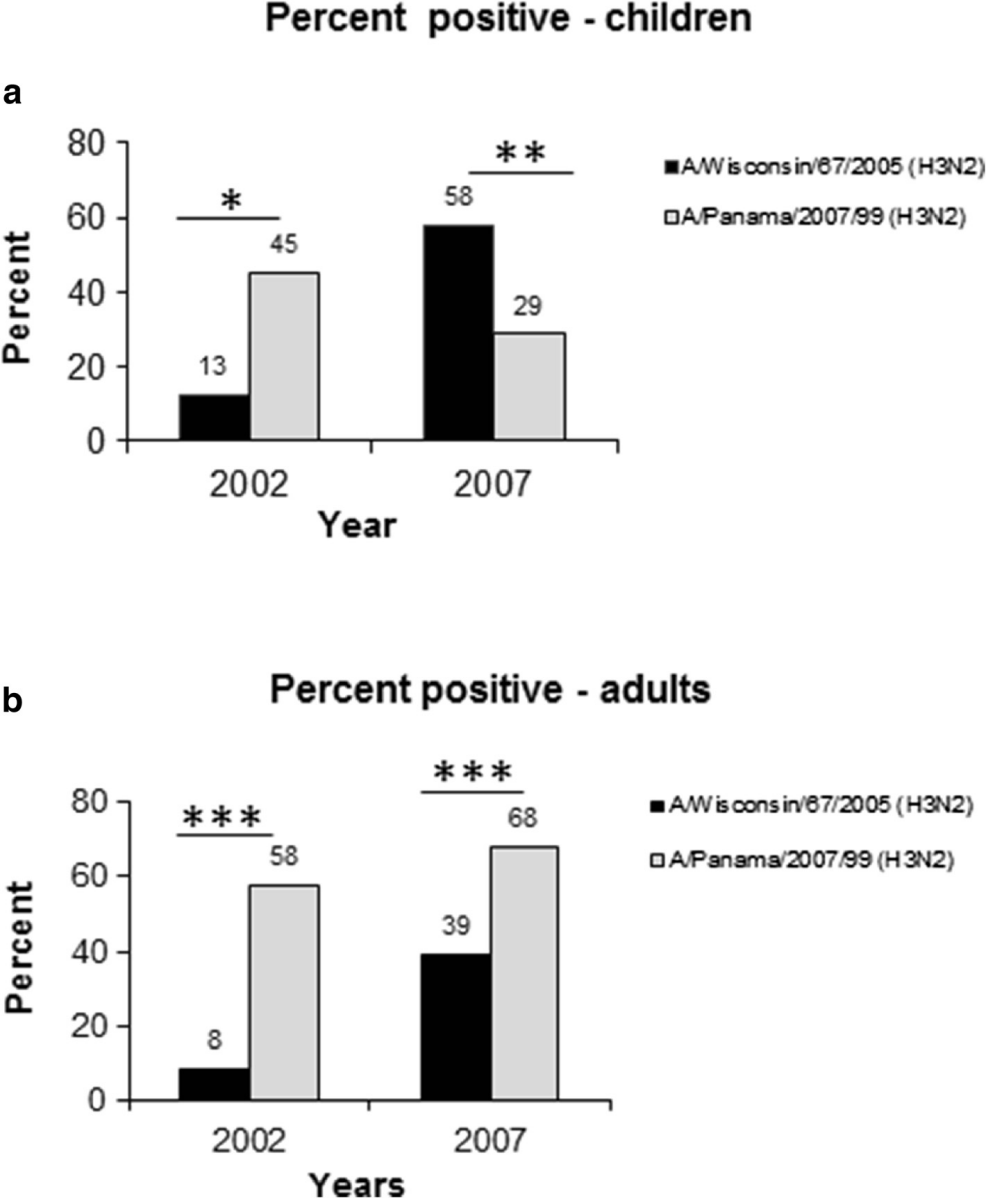
**Anti-influenza antibodies in young children and in young adults, 2002 and 2007.** Children (1-3-years-old, **a**) and adult (35-50-years-old, **b**) sera obtained in 2002 and in 2007 were tested by the HI test against A/Wisconsin/67/2005 (black columns), and A/Panama/2007/99 (gray columns). * <0.0013, ** <0.01, *** <0.0001 using Chi test.

**Figure 3:**
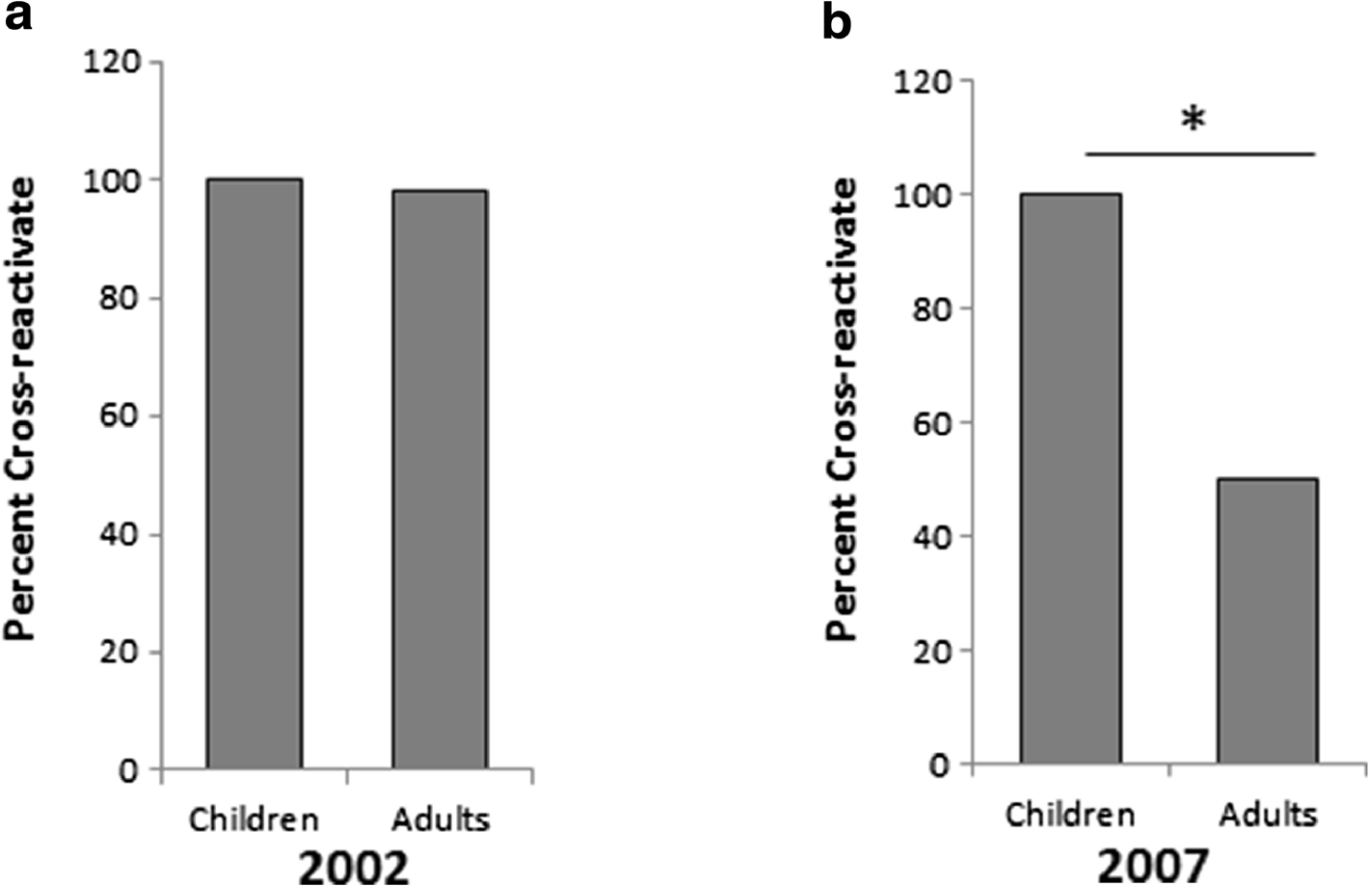
**Percentages of antibody cross-reactivity.** Percentages of antibody cross-reactivity in 2002 **(a)** and in 2007 **(b)**. In 2002, antibodies against A/Wisconsin/67/05 were set to be 100% and the anti-A/Panama/2007/99 were compared to them, while in 2007, the A/Panama/2007/99 antibodies were set to be 100% and were compared with the A/Wisconsin/67/05 antibodies. * <0.0001 using Chi test.

The significance of the differences in the percentages of sera containing antibodies in Figures 1 and 2 were calculated using the Chi test. A p value of <0.05 was considered statistically significant. Student paired *T*-test was used in Figure 3 and a P value of <0.05 was considered statistically significant.

## Results

### Influenza strains circulating in Israel between 1998–9 and 2007–8

Since 1996, the ICDC has operated a network of sentinel community clinics in Israel, during the winter months. Swabs from patients with influenza-like illness are transported to the National Influenza Center at the Central Virology Laboratory at the Ministry of Health, for virus identification, isolation and typing. A description of the H3N2 viruses isolated between 1999 and 2009 are shown in Table 1 and a phylogenetic tree of all viruses is presented in Figure 1.

**Table 1:** Influenza viruses circulating in the Israeli population between 1998-2008

	**Year**	**Circulating strains A(H3N2)**
	1998-1999	**A/Sydney/5/97-like**
	1999-2000	**A/Sydney/5/97-like**
	2000-2001	none
Testing sera ➞	2001-2002	**A/Panama/2007/99-like**
	2002-2003	A/Panama/2007/99-like
	2003-2004	A/Fujian/411/02-like*
	2004-2005	**A/Wyoming/3/03-like**
	2005-2006	A/California/7/04-like
Testing sera ➞	2006-2007	**A/Wisconsin/67/05-like**^**#**^
	2007-2008	A/Brisbane/10/07-like
	2008-2009	A/Brisbane/10/07-like

*Not include in the influenza vaccine for the same year.

^#^About 1/3 of the isolates had reduced titers against this strain.

In bold- the dominant circulating strain.

Until the summer of 2002, the dominant H3N2 viruses were either A/Panama/2007/99 or the closely related virus A/Sydney/5/97 (Figure 1 and [21]). In the winter of 2002–2003, only a few cases of A/Panama/2007/99 infections were observed (Table 1). The 2003–2004 year was dominated by the new A/Fujian/411/02 H3N2 subtype strain, which is an antigenic drift of the A/Panama/2007/99 virus (Figure 1 and [21,22]). In the 2004–2005 season A/Wyoming/3/03, which is antigenically similar to A/Fujian/411/02 [23], circulated in the country. In 2005–2006 the H3N2 virus A/California/7/04-like virus was not the dominant virus. In the winter season of 2006–2007, the slightly different A/Wisconsin/67/05 H3N2 virus strain [22] circulated in Israel (Figure 1). Therefore, we decided to investigate the presence of anti-influenza antibodies against A/Panama/2007/99 in samples collected in the summer of 2002, following several years in which the virus strain, or others similar to it, were present in the country (Figure 1 and Table 1). An identical analysis was performed on samples of the summer of 2007, following four years in which A/Fujian/411/02 or similar viruses were present (Figure 1 and Table 1).

### Anti-influenza antibodies in children

To test for the presence of anti-influenza antibodies in the population, 400 randomly selected sera samples from adults aged 30–50 and 80 samples from children aged 1–3 were obtained for each of the years 2002 and 2007. Samples were selected from those collected between April and November, when the influenza virus is not usually active in the country. Importantly, it’s necessary to clarity whether the children that evaluated in this manuscript were vaccinated against influenza. To verify this, we tested the sera of the children for recognition of the H1N1 virus (A/New Caledonia/20/1999) that was applied in the vaccine given at each particular season and also for the recognition of the influenza B viruses present in the vaccine (B/Sichuan/379/99, in 2001–2002 and B/Malasia/2506/04 in 2006–2007). All children were negative for at least one of the viruses present in the vaccine strains indicating that they were not vaccinated.

As can be seen in Figure 2a, in the summer of 2002, 45% of the sera derived from children had antibodies against the influenza strain A/Panama/2007/99 (H3N2), a strain which had circulated in the population in the preceding winters (Table 1). It is therefore likely that the tested children were indeed exposed to these viruses. However, surprisingly, 13% of the children had antibodies against A/Wisconsin/67/2005 (H3N2), a virus that was not isolated in Israel prior to 2006.

In 2007, 58% of the tested children had antibodies against A/Wisconsin/67/05 (H3N2) (the dominant strain in the winter of 2006–7). Unexpectedly, 29% of the sera obtained from children under the age of 3 in 2007, showed reactivity against A/Panama/2007/99 (H3N2), although this strain was not detected in the population after 2003.

### Anti-influenza antibodies in adults

In 2002, the antibody distribution detected in sera from adults was similar to that obtained in children, with 58% of the adults exhibiting antibodies directed against A/Panama/2007/99 (Figure 2b). In 2007, however, the results in adults differed from the findings in children. A high percentage (68%) of healthy adults had antibodies directed against A/Panama/2007/99, a virus which was not detected in the Israeli population after 2003. Thirty-nine percent of the adults had antibodies directed against A/Wisconsin/67/05, the strain which had been dominant in the preceding winter.

### Cross-reactivity among various anti-influenza antibodies

Since antibodies were detected against virus strains to which the children had never been exposed, both in 2002 and in 2007, we speculated that the antibodies are cross-reactive. Strikingly, the anti-A/Wisconsin/67/05 antibodies that were detected in 2002 in 13% of the children samples were only detected in children who also had antibodies to A/Panama/2007/99 (Figure 3). These findings suggest a cross–reactivity of the antibodies to these two virus strains, as these children had not been exposed to the former virus. In 2007, all of the 29% children that had antibodies against A/Panama/2007/99 (Figure 2) also had antibodies to A/Wisconsin/67/05 (Figure 3). The oldest children that were evaluated in 2007 were born in 2004, one year after the A/Panama/2007/99 strain was last documented in Israel, suggesting that the antibody recognition of A/Panama/2007/99 observed in children in 2007 was also due to cross-reactivity.In contrast, in adults, in 2007, 50% of the anti-A/Panama/2007/99 antibodies cross-reacted with A/Wisconsin/67/05, while in 2002 the majority of of the adults (out of the 8%, Figure 2) that had antibodies against A/Panama also had antibodies against A/Wisconsin (Figure 3). We thus concluded that while the antibody repertoire detected in adults was likely to reflect the various influenza virus strains to which they were exposed to during their life, in children, it seemingly represents “true” cross-reactive antibodies.

### Titers of anti-influenza antibodies

**Figure 4:**
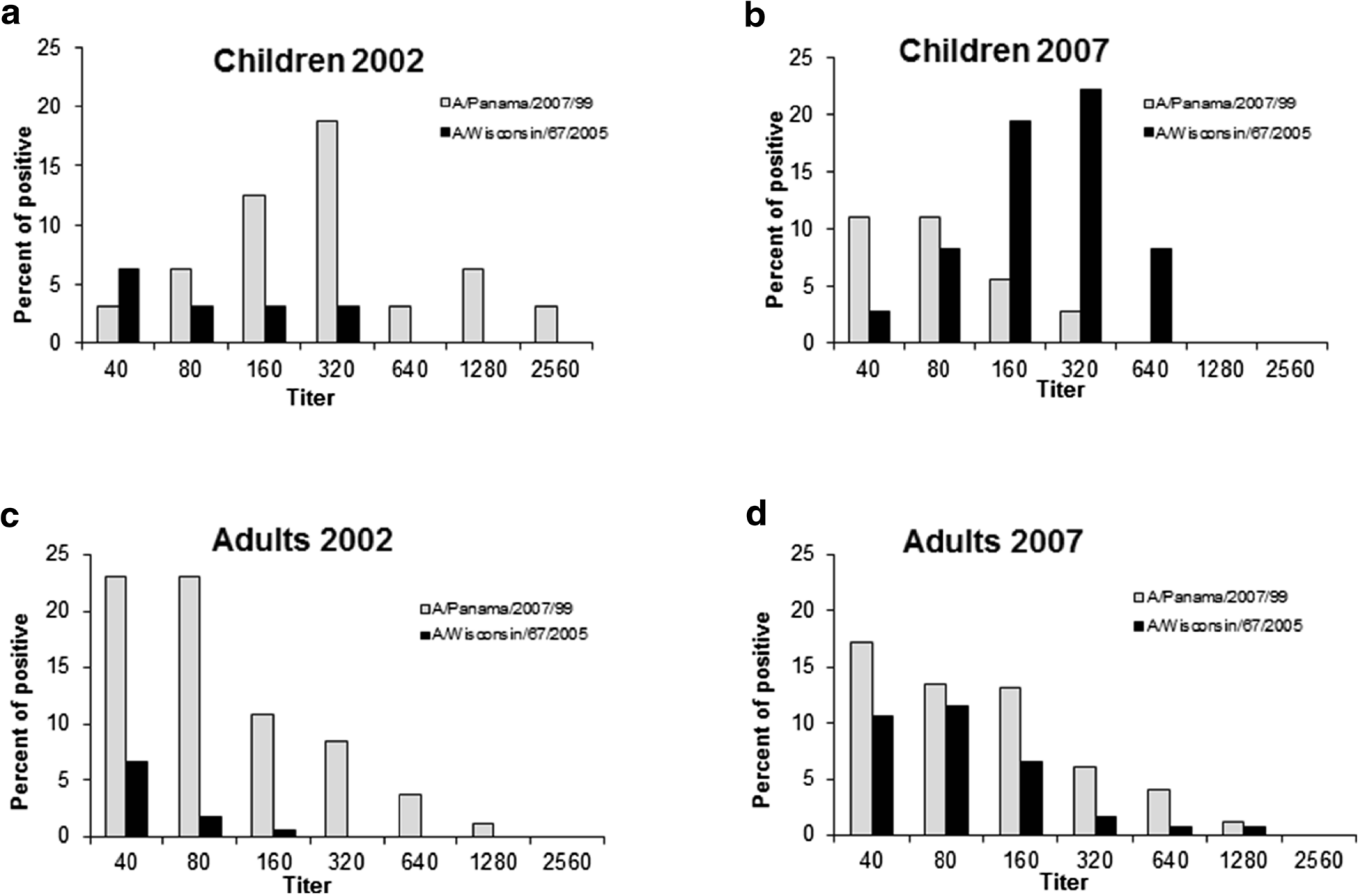
**Distribution of antibody titers in young children and in young adults, 2002 and 2007.** Antibody titers in children **(a and b)** and in adults **(c and d)** in 2002 **(a and c)** and 2007 **(b and d)** were determined using the HI test.

In attempt to understand how antibodies against a particular strain were detected in the population when the virus had been absent, we examined the titers of the anti-influenza antibodies in children and adults, as an estimation of the effectiveness and the strength of the immune response (Figure 4). In 2002, the highest antibody titers observed both in adults and in children were directed against A/Panama/2007/99. In contrast, in 2007, the highest antibody titers observed in children were directed against A/Wisconsin/67/05, the dominant strain in the preceding winter. In adults, however, the highest 2007 antibody titers were against A/Panama/2007/99, while A/Wisconsin/67/05 was recognized to a slightly lesser extent, likely due to repeated exposure to A/Panama/2007/99-like viruses in previous years (Figure 4b and d).Moreover, in children, the distribution of antibody titers observed against A/Panama/2007/99 and against A/Wisconsin/67/05, in 2002 and in 2007, respectively, resembled a bell-like shape (Figure 4a and b), supporting the assumption that the children were indeed infected by the viruses. In contrast, in adults, bell-like graphs were not observed, probably because the adult sera contains antibodies directed against several influenza virus strains that they had been exposed to over the years (Figure 4).

## Discussion and conclusions

In this study, we evaluated the anti-influenza antibodies detected in the Israeli toddlers and adult population in the summer seasons of 2002 and 2007, following a marked antigenic drift in the influenza virus. We tested the 2002 anti-influenza antibodies in samples collected after a period that was dominated by a single H3N2 strain, A/Panama/2007/99, as well as samples collected at the close of 2007, following four years that were dominated by a markedly different H3N2 viruses (as seen in our phylogenetic tree and as acknowledged previously, [22,24,25]). We demonstrated that a significant percentage of 1-3-year-old children with naturally occurring antibodies against the currently circulating strains, also had antibodies against markedly different strains to which they had never been exposed. Of note, the children whose samples were studied here, had not been vaccinated/immunized against influenza and thus the cross-reactive antibodies observed in the hospitalized patients could not be associated with vaccination. Overall, substantial antibody cross-reactivity against seasonal influenza virus strains was seen in this population.

To the best of our knowledge, the presence of naturally occurring cross-reactive antibodies against seasonal infections following significant antigenic drift has not been reported. It has been suggested that some elderly people likely to have been exposed to the 1918 pandemic influenza infection, bear cross-reactive antibodies against the pandemic 2009 swine origin influenza virus [26]. However, cross-reactive antibodies against seasonal influenza infections were not observed [26].

The results of the current study can contribute to the understanding of the epidemiology of influenza, the elicited immune responses and the transmission rates. For example, a recent survey testing approximately 12,000 individuals for antibodies against the avian H5N1 virus, demonstrated a much higher percentage of the population with the antibodies than previously estimated [27]. The authors concluded that this virus, considered highly dangerous (60% mortality), can also cause mild or subclinical infections. However, it is possible that some or all of the antibodies detected were cross-reactions with other circulating influenza viruses.

It is difficult to assay for antibody cross-reactivity in adults, since it is impossible to trace the exact strains an individual was exposed to during his life. One of the strengths of the current study was the use of sera from young children that had not previously been exposed to the tested virus stains. Thus, if the sera contained antibodies directed against influenza strains that were not circulating within their short life, a phenomenon of antibody cross-reactivity is highly likely.

We have shown that the anti-influenza antibodies directed against A/Panama/2007/99 and against A/Wisconsin/67/05 cross-react with each other. The assays that were performed to test the presence of the anti-influenza antibodies were hemagglutination inhibition tests. In these types of assays, a requirement of 40 HI units defines a protective antibody [13]; thus, these cross-reactive antibodies might provide protective functions.

Interestingly, not all antibodies cross-reacted with each other. One possible way to explain this is that in all cases cross-reactive antibodies are generated but we did not detect these in our assays. Regardless of whether this is accurate or not, it is clear that protective cross-reactive antibodies were probably not generated in every individual. It is possible that the genetic background of the individuals dictated whether he will develop cross-reactive antibodies. In this regard, it will be interesting to characterize the MHC class I and class II haplotypes of individuals who generated cross-reactive protective antibodies and to compare to the MHC classes of those who did not.

Antibody cross-reactivity has been previously reported following vaccination against influenza [28]. In addition, the presence of cross-reactive and vaccine-induced antibodies to the newly emerging virus swine influenza A(H3N2) was also recently demonstrated [29].

Furthermore, several cross-reactive monoclonal antibodies that cross-react with various influenza A and influenza B viruses developed following immunization. These antibodies recognize distinct conserved epitopes in the head region of the hemagglutinin derived from influenza A and B viruses [30]. Finally, it was also reported that more cross-reactive antibodies are generated following infection when compared with the titers detected following vaccination [31]. Thus, understanding the mechanisms leading to antibody cross-reactivity could provide information essential for the design of broadly, cross-reactive vaccine.

In vivo studies have been conducted to evaluate the efficiency of cross-reactive antibodies in vivo. Studies in mice and in ferrets have shown that classical swine H1N1 influenza viruses confer cross-protection against swine-origin 2009 pandemic H1N1 influenza virus infection [32]. The broadly cross-reactive antibodies mentioned above effectively protected against lethal challenge with influenza A and B viruses [30]. The presence of naturally occurring cross-reactive antibodies against markedly different influenza virus strains, in a significant percentage of children, suggests that the in vivo cross-reactive experiments performed have human relevancy. Furthermore, our results suggest that development of a broad reactive anti-influenza vaccine may be feasible.

## Competing interests

The authors declare that they have no competing interest.

## Authors’ contributions

MM, MB, HS, IZ, KY, RB, reviewed and collected the data. MM and MG conceived the study and wrote the paper. RD, was responsible for statistical analysis. DC, TS, EM supervised the work and reviewed the manuscript drafted the manuscript. All authors reviewed the work and approved the final manuscript.

## Pre-publication history

The pre-publication history for this paper can be accessed here:

http://www.biomedcentral.com/1471-2334/14/346/prepub
